# Red Ginseng Is a Therapeutic Candidate for Chronic Doxorubicin-Induced Cardiomyopathy in Mice

**DOI:** 10.1155/2023/4085409

**Published:** 2023-11-30

**Authors:** Naoki Yoshikawa, Naoto Hirata, Yuichiro Kurone, Shin Ohta, Sadahiko Shimoeda

**Affiliations:** Department of Pharmaceutical Health Care and Sciences, Tokyo University of Pharmacy and Life Sciences, 1432-1 Horinouchi, Hachioji, Tokyo 192-0392, Japan

## Abstract

Doxorubicin-induced cardiomyopathy (DICM) is associated with a poor prognosis, and effective therapeutic drug candidates have yet to be identified. Furthermore, whether basic animal models reflect the clinical pathogenesis of DICM should be carefully examined. Although the exact mechanisms underlying the development of DICM are complex and remain unclear, oxidative stress is strongly implicated as a contributing factor. Therefore, we investigated the effects of ginseng (the root of *Panax ginseng*: Gin), an inexpensive and safe drug with antioxidant properties. We previously conducted a meta-analysis that yielded results suggesting its efficacy in humans. However, this study did not examine the efficacy of ginseng in detail. Therefore, this study investigated the efficacy of red ginseng (steamed and dried ginseng cultivated for over six years; RGin) in a mouse model of chronic DICM to elucidate its potential therapeutic benefits. RGin prevented the decrease in left ventricular ejection fraction associated with doxorubicin (DXR) administration and prolonged survival in DBA/2 mice. In addition, RGin reduced DXR-induced cardiomyocyte damage. These findings highlight its potential as a therapeutic option for the treatment of DICM.

## 1. Introduction

Cancer survivors have experienced new complications in recent years due to prolonged treatment. One complication is cancer therapy-related cardiac dysfunction (CTRCD) [1]. Doxorubicin (DXR), a well-established chemotherapeutic agent, causes doxorubicin-induced cardiomyopathy (DICM) [2]. DICM has a poorer prognosis than in ischemic heart disease [3]. DICM must be managed separately from other cardiac diseases because it has a poor prognosis compared to that of other cardiac diseases, and its pathogenesis has not been established. Many characteristic pathological findings exist, such as severe dilatation of the atria, ventricles, and adria cells, and at least two pathologies were available based on onset time [4]. The first is the acute toxicity of DXR, which occurs within a few hours of DXR administration and is not dose-dependent. The acute phase presents mainly with electrocardiographic abnormalities and is mostly transient [5]. Second, chronic DXR toxicity is characterized by a decrease in cardiac function that develops months to decades after drug administration. Furthermore, DICM causes dose-dependent and irreversible pathology [1, 6].

The incidence of DICM was previously thought to rapidly increase at doses >500 mg/m^2^. However, recent guidelines consider a cumulative DXR dose >250 mg/m^2^ to be a high-risk factor for the onset of DICM [6]. However, many reported studies have been conducted in acute toxicity models constructed by administering high doses of doxorubicin in single or frequent short-term doses. In addition, many previous reports may not align with clinical problems [7]. Furthermore, the mechanisms underlying DICM pathogenesis involve a complex interplay among all pathways, including reactive oxygen species (ROS) generation, apoptosis, and autophagy [8, 9]. Although there is still insufficient consensus on the detailed etiology of DICM, the involvement of ROS is strongly supported [10]. DXR generates ROS because it has a high affinity for all tissues and can easily enter cells. When DXR enters the cell, its characteristic quinone structure receives electrons from NADPH. Thus, it is converted into a reduced and unstable DXR molecule with a semiquinone structure [11]. The unstable DXR reacts with oxygen to promote ROS generation. In addition, reduced DXR is restored to stable DXR through a reaction with Fe^3+^ to produce Fe^2+^, which promotes the Fenton reaction required for ROS production [12]. The oxidative reduction reactions caused by several factors, with DXR as the central molecule, are cyclically accelerated. This resulted in the generation of abundant ROS from a single DXR molecule. This is one of the most supported hypotheses for the pathogenesis of DICM [13, 14].

Therefore, searching for effective and safe agents that can reduce DXR toxicity and inhibit DICM progression remains crucial. Established treatment options for DICM are limited. Therefore, we investigated the antioxidant effects of ginseng (Gin), a low-cost, safe drug with antioxidant activity. Gin administration to patients undergoing anthracycline chemotherapy inhibits the reduction in left ventricular ejection fraction (LVEF) [15]. However, this meta-analysis did not comprehensively examine the efficacy of ginseng.

Therefore, this study aimed to investigate the effects of intraperitoneal administration of red ginseng (RGin) in a chronic DICM mouse model. RGin is a processed form of Gin that has augmented antioxidant effects, and it is commonly used as a medication in several East Asian countries [16].

## 2. Materials and Methods

### 2.1. Animals

Four-week-old male ICR (*n* = 30) and DBA/2 (*n* = 60) mice were obtained from Japan SLC Inc. All mice were acclimated in a pathogen-free animal facility for six days after purchase. Each mouse was randomly assigned to one of three groups: normal saline (NS) (*n* = 10), DXR (*n* = 10), or DXR + RGin (*n* = 10). The same three groups of DBA2 mice were used for additional investigations. All mice were maintained on a 12-h light/dark cycle and had ad libitum access to food and water.

### 2.2. Ethics

All experiments and animal care were conducted following the principles of Good Laboratory Practice. The experimental animals were handled and treated in strict compliance with the regulations for handling experimental animals at the Tokyo University of Pharmacy and Life Sciences. This study was approved by the Experimental Animal Committee (Permit No.: P19-62).

### 2.3. Drug Administration Protocol

DXR (4 mg/kg) was administered intraperitoneally on days 2, 9, 16, 25, and 32 at a cumulative dose of 20 mg/kg. Similarly, RGin (5 g/kg) was intraperitoneally administered three times a week for a total of 15 days (days 1, 3, 5, 8, 10, 12,…, 31, 33, and 35). NS was administered at the same volume as the saline solution of DXR or RGin at the corresponding time points.

### 2.4. Drugs

DXR (10 mg/5 mL) was purchased from Sandoz Co. (Basel, Switzerland). The RGin injection was formulated using ginseng powder purchased from Tsumura Co. (Tokyo, Japan). The saline solution was purchased from Otsuka Pharmaceutical Co. (Tokyo, Japan). Ethanol (99.5% purity), butorphanol, medetomidine, and midazolam were purchased from Fujifilm Wako Pure Chemical Co. (Osaka, Japan).

### 2.5. Sample Collection

The sample collection protocol complied with the guidelines and regulations of the Animal Experiment Committee. Echocardiography examinations were performed under general anesthesia using a combination of butorphanol (5 mg/kg), medetomidine (0.75 mg/kg), and midazolam (4 mg/kg). Mice were euthanized using carbon dioxide gas after echocardiography. After that, the abdomen was opened using dissecting scissors, and total blood was collected from the inferior vena cava for biomarker measurements. Plasma was collected by centrifugation at 1200 × *g* for 20 min, frozen at −80°C, and thawed to room temperature at the time of measurement. In addition, hearts were collected for tissue evaluation at week 7.

### 2.6. Preparation of a RGin Injection Solution

RGin powder (5 g) was dissolved in 50% ethanol (100 mL). The mixture was boiled until it was reduced by half, and the extract was purified. The extract was subsequently centrifuged at 2150 rpm for 45 min using a low-speed refrigerated AX-501 centrifuge (Tomy Digital Biology Co., Ltd., Tokyo, Japan), and the supernatants were carefully separated. The supernatant was freeze-dried and reconstituted by adding 10 mL of saline solution to the dried material and filtering it before use. The resulting solution with a concentration of 500 mg/mL RGin was used for RGin injection. The RGin powder was sourced from a single lot with its uniformity reasonably assured by the pharmaceutical company. The extraction process was repeated five times to obtain five separate extraction lots. They were combined and lyophilized to produce large amounts of homogenized, lyophilized material. A portion of the lyophilized material prepared using our extraction method was subjected to quantitative analysis using liquid chromatography with tandem mass spectrometry (LC-MS/MS). The results revealed the presence of representative ginsenosides Rb1, Rg1, and Rg3 (S) at levels of 5.3 mg/g, 4.6 mg/g, and 0.21 mg/g, respectively.

### 2.7. Echocardiography

Echocardiographic examinations were performed at 1-week and 7-week time points. Butorphanol, medetomidine, and midazolam were intraperitoneally administered to mice under general anesthesia. Unnecessary chest hair was removed using an electric shaver after confirming the disappearance of the ortho-rectal reflex. An M-mode echocardiogram was obtained using a Sonoscape SV6 7-4 MHz probe (SonoScape Medical Corp., Shenzhen, China). The left ventricle in the image was assumed to be a rotating ellipsoid. The vertical distance from the endocardial surface of the left ventricular wall to that of the posterior wall of the left ventricle was measured on the short-axis image in the beam direction, passing through the largest short diameter of the left ventricle at end-diastole and end-systole. The end-diastolic (left ventricular end-diastolic diameter; LVDd) and end-systolic (LVD) diameters were determined based on these measurements. LVEF was calculated using the Teichholz method. This method involves calculating the left ventricular end-diastolic volume (LVEDV) and left ventricular end-systolic volume (LVESV) and then deriving FS (%) and LVEF (%) as follows:

(1) $$\begin{array}{c} \text{FS }\left(\%\right)\;=\frac{\left(\text{LVDd }‐\text{ LVDs}\right)}{\text{LVDd }}\;\times 100,\\ \text{LVEF }\left(\%\right)\;=\frac{\left(\text{LVEDV }‐\text{ LVESV}\right)}{\text{LVEDV}}\;\times 100. \end{array}$$

### 2.8. Survival Analysis

Survival was monitored after five weeks of drug administration for up to seven weeks (12 weeks). The occurrence of an event was defined as death and was evaluated by recording the number of mice that died and the number of survival days.

### 2.9. Quantitative Measurement of NT-proBNP and Cardiac Troponin I (cTnI)

Assay kits for NT-proBNP (LS-Bio, Lynnwood, WA, USA) and cTnI (Life Diagnostics Inc., West Chester, PA, USA) were used for biomarker measurements. All samples were assayed at least in duplicate according to the manufacturer's instructions. NT-proBNP is an N-terminal peptide chain produced from the precursor pro-BNP in equal amounts as brain natriuretic peptide (BNP), and it shows no bioactivity; however, it is more stable *in vivo* and has a longer half-life than in BNP. Therefore, it is commonly used as a stable marker for heart failure.

### 2.10. Histological Evaluation through Hematoxylin and Eosin (HE) Staining

The hearts were washed with saline and immediately fixed in a 10% neutral-buffered formalin solution (pH 7.4) after appropriate removal and separation using dissecting scissors. Fixed tissues were transported to an external laboratory (Biopathology Institute Co., Ltd., Oita, Japan) and embedded in paraffin. Tissues excised from the aortic valve were stained with HE. Histopathological evaluation was performed using Olympus Net Image Server SQL (Olympus Co., Ltd., Tokyo, Japan).

### 2.11. Statistical Analyses

All data were analyzed using Easy R (EZR) version 1.55 [17]. Between-group comparisons were performed using a one-way analysis of variance (ANOVA) and analyzed using a post hoc Tukey test. Survival functions were estimated using the Kaplan–Meier method and compared using the log-rank test. Statistical significance was set at *P*  < 0.05 in all analyses.

## 3. Results

### 3.1. The Effect of RGin on the Survival of DXR-Treated Mice

**Figure 1:**
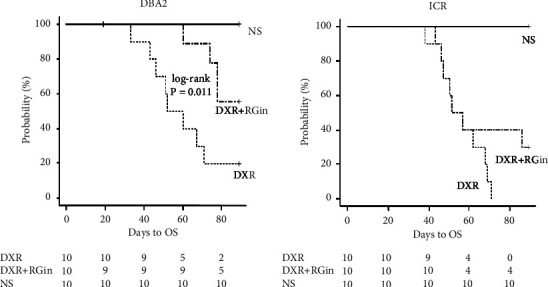
Survival duration curves after drug administration in DBA/2 and ICR mice: (a) Kaplan–Meier curves in DBA/2 mice and (b) Kaplan–Meier curves in ICR mice.

DICM is a serious disease with a poor prognosis, and improved survival is a desirable outcome. ICR and DBA/2 mice were used in this study. This revealed an increased mortality rate starting on day 40 after DXR administration in both strains. DBA/2 mice showed a favorable response to RGin treatment (Figure 1(a)), whereas ICR mice did not exhibit the same effect (Figure 1(b)). Moreover, a significantly prolonged survival duration was observed in the DXR + RG group of DBA/2 mice (*P*=0.011). However, a trend similar to that in the DXR group emerged from day 60 onwards and was characterized by an increase in mortality rates. Animals that survived until the end of the observation period were euthanized to minimize suffering.

### 3.2. Protective Effects of RGin on DICM in DBA/2 Mice

**Figure 2:**
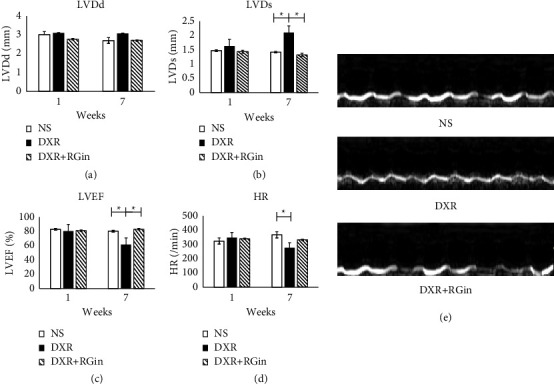
Evaluation of cardiac function using echocardiography. (a) LVDd, (b) LVDs, (c) LVEF, (d) HR, and (e) M-mode echocardiographic images showing cardiac function. ^*∗*^*P*  < 0.05 was considered a significant difference. LVDd, left ventricular end-diastolic diameter; LVDs, left ventricular end-systolic diameters; LVEF, left ventricular ejection fraction; HR, heart rate.

DICM is clinically diagnosed based on decreased LVEF. Therefore, monitoring LVEF over time is crucial for basic research. Echocardiography was performed on all animals at weeks 1 and 7. A statistically significant decrease was observed in LVEF in the DXR group compared to that in the NS and DXR + RG + RG groups (Figure 2(c)). In contrast, the DRX + RG group had higher LVEF levels. These results indicated that RGin effectively prevented DXR-induced decline in cardiac function.

### 3.3. The Effect of RGin on NT-proBNP and cTnI in DXR-Treated DBA/2 Mice

**Figure 3:**
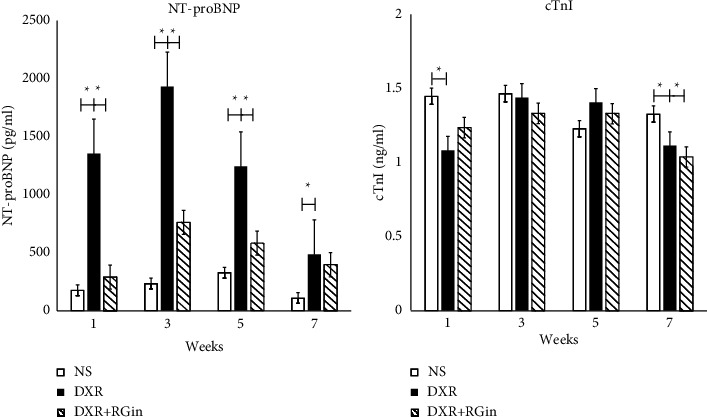
NT-proBNP and cTnI were measured using ELISA. (a) Plasma concentration of NT-proBNP. (b) Plasma concentration of cTnI. ^*∗*^*P*  < 0.05 was considered significantly different.

NT-proBNP is an established marker of heart failure and is used to assess cardiac load and fluid retention. In the DXR group, NT-proBNP levels increased significantly immediately after drug administration compared with those in the NS group throughout the observation period (Figure 3(a)). In contrast, RGin administration significantly suppressed the increase in NT-proBNP levels induced by DXR in the DXR + RG + RG group (weeks 1–5). However, caution must be exercised when interpreting these findings, as there was a trend toward increased NT-proBNP levels compared with those in the NS group despite the lack of statistically significant differences. Furthermore, there was no significant difference compared to those in the DXR group at week 7.

The levels of cTnI, a biomarker of cardiomyocyte damage, did not increase after DXR administration (Figure 3(b)). The results showed unexpected findings, such as lower levels than those in the NS group. Consistent data were not observed in any group or measurement point.

### 3.4. Evaluation of RGin Administration on Cardiomyocytes Using HE Staining

**Figure 4:**
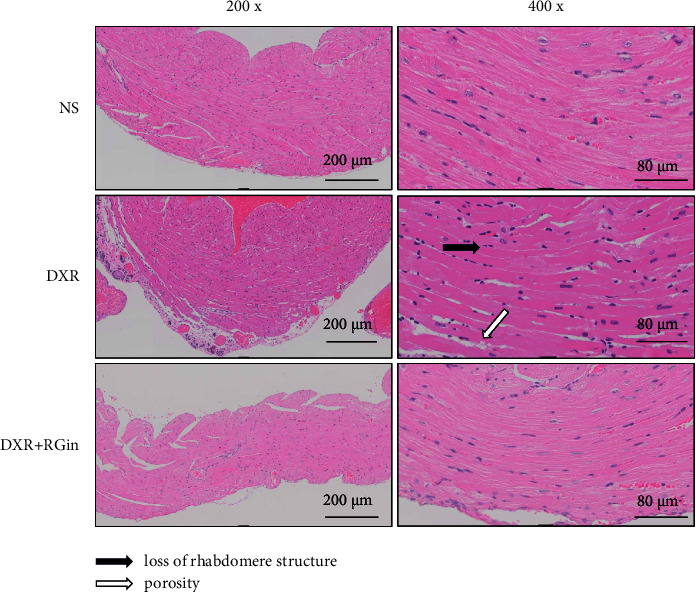
HE staining in cardiomyocytes of DBA/2 mice at week 7 (×200, 400).

Hematoxylin and eosin-stained images of cardiomyocytes obtained from myocardial tissue at week 7 revealed various abnormal findings in the DXR group, including loss of rhabdomere structure, hypertrophy, intense staining, and vacuolation (Figure 4). Although these findings are not specific to DICM, they are consistent with previous reports. In contrast, the DXR + RG + RG group showed a lower incidence of these abnormal findings than that in the DXR group, suggesting a potential protective effect of RGin in cardiomyocytes.

## 4. Discussion

Our previous study extensively explored the efficacy of Kampo medicine in the treatment of DICM (15). Kampo medicinal formulations containing Gin were effective against DICM. Although a few of our studies involved the intravenous injection of Kampo medicine, this method is not commonly used. However, recent reports have highlighted the potential benefits of injecting Kampo medicine to improve the prognosis of conditions such as acute-phase sepsis. Thus, in recent years, interest in the efficacy of herbal medicines for all pathological conditions has increased, regardless of the route of administration [18]. Therefore, this study aimed to investigate the efficacy of RGin in a mouse model of chronic DICM. Here, the intraperitoneal injection of RGin into DBA/2 mice reduced the cardiotoxicity of DXR. This demonstrates that the combined administration of DXR and RGin preserves LVEF at a high level, alleviates excessive load, suppresses myocardial impairment, and extends survival. These findings are consistent with those of our previous study [15].

DBA/2 and ICR mice were used in this study. DBA/2 mice show a high incidence of myocardial calcification. Furthermore, they show high reactivity with the water-soluble fraction derived from the culture supernatant of *Candida albicans* (*Candida albicans* water-soluble fraction; CAWS) and are prone to vasculitis [19, 20]. Therefore, we hypothesized that DBA/2 mice would be susceptible to cardiotoxicity. However, the survival curves showed that ICR and DBA/2 mice showed similar reactivity to DXR toxicity. In contrast, the therapeutic effect of RGin was promising only in DBA/2 mice. Based on these results, we investigated these effects in DBA/2 mice. The long-term survival curves of the DXR + RG group exhibited a similar trend to that of the DXR group. This suggests that the effectiveness of RGin is temporary and does not completely inhibit DXR-induced cardiotoxicity. The discontinuation of RGin administration after 5 weeks in this study is a limitation, as the long-term therapeutic effects of RGin could not be fully clarified. ICR mice may exhibit high sensitivity even at low doses of DXR [21]. Alternatively, the DBA/2 mice may exhibit resistance to DXR toxicity. However, we are unable to provide sufficient information at this point. Numerous basic studies with standardized DXR doses and sufficient observation periods are required to determine the differences between animal strains. Although chronic DICM is a clinical concern, many basic studies have used acute DICM models that do not provide a consistent foundation for our discussion.

However, the behavior of cTnI remains unclear. This structural protein is found in muscle fibers, including the myocardium, and has been clinically established as a biomarker for conditions such as myocardial infarction and acute coronary syndrome. However, the cut-off value for cTnI may vary depending on the facility and measurement method used. In addition, the utility of cTnI in DICM has not been fully established [22–25]. Cardinale previously showed that early and/or sustained troponin elevation in patients receiving DXR correlated with the incidence of future cardiovascular events [26]. Therefore, we conducted blood sampling 48 h (1–5 weeks) or 14 days (7 weeks) after DXR administration. However, our results did not show significant variations in cTnI levels. Although a few studies have investigated troponin as an indicator of DICM in mouse models, data on the continuous monitoring of cTnI during long-term administration of low-dose DXR are limited [27, 28]. cTnI levels in mice stimulated by another drug peak immediately after exposure decreased to baseline levels within approximately 48 h [29]. Therefore, the cTnI peak may have been missed in this study. Thus, careful consideration of the sampling time is necessary to accurately evaluate cTnI levels in DICM mice.

RGin contains various active components, including saponins, polysaccharides, and essential oils [30]. Ginsenosides, a type of saponin, exhibit pharmacological effects [31]. Ginsenosides are rich in the following subtypes: Rg1, Rg3, and Rh2, which act as cardioprotective compounds against DICM [32–34]. Although the dose of DXR administered differed, the results consistently indicated that these ginsenosides inhibited DICM. These reports differ from ours in which they report the efficacy of specific ginsenosides isolated from ginseng. For example, Wang et al. reported that the administration of ginsenoside Rg3 to animals suppressed DICM, and our results were consistent with these findings [32]. However, the high-fat solubility of ginsenoside Rg3 must be addressed for formulation purposes [33]. However, the mechanisms underlying the action of RGin in DICM have not yet been sufficiently established. The most likely mechanisms of action are believed to be antioxidants and antiapoptotics. Recent studies have suggested that the activation of the Nrf2 pathway is involved in these effects. Wang et al. concluded that ginsenoside Rg3 is effective against DICM and that its mechanism involves the alleviation of oxidative stress through Nrf2 pathway activation [32]. Zhe et al. reported the effectiveness of ginsenoside Rg1 against DICM by improving the Bcl-2/Bax ratio, inhibiting caspase release, and improving apoptosis [35]. Using the same ginsenoside Rg1, Xu showed the potential for DICM improvement by inhibiting endoplasmic reticulum stress and autophagy [36]. However, all these reports used mouse models of acute DICM. Furthermore, it is important to note that there is currently no consensus regarding the effective components, dosages, or the specific mechanism of action of RGin in the context of DICM.

In this study, we investigated the drug repositioning of a common RGin powder extract without isolating specific ginsenosides. These results demonstrated effective therapeutic potential against DICM. Although no drug currently isolates specific ginsenosides, many fundamental studies have focused on individual ginsenosides for investigation. This report highlights the need to develop agents that can isolate specific ginsenosides and accelerate drug development for treating DICM in clinical practice. However, our study has certain limitations. First, we did not explore all the active ingredients in RGin. Second, we analyzed the components of the extract; however, we were unable to identify the specific active ingredients. Third, the study results were based on a single dose. Therefore, the findings should be interpreted with caution.

## 5. Conclusions

We propose that RGin extract, which is not limited to specific compounds, is a promising therapeutic candidate for chronic DICM. This hypothesis is strongly supported by the evidence demonstrating its ability to prolong survival and preserve cardiac function.

## Data Availability

The data used to support our findings of this study are included in this article.
